# Diversity and Role of Prophages in *Pseudomonas aeruginosa*: Resistance Genes and Bacterial Interactions

**DOI:** 10.3390/genes16060656

**Published:** 2025-05-29

**Authors:** Keyla Vitória Marques Xavier, Adrianne Maria de Albuquerque Silva, Ana Carolina de Oliveira Luz, Felipe Santana Caboclo da Silva, Beatriz Souza Toscano de Melo, João Luiz de Lemos Padilha Pitta, Tereza Cristina Leal-Balbino

**Affiliations:** 1 Department of Microbiology, Aggeu Magalhães Institute, IAM/Fiocruz, Recife 50740-465, PE, Brazil; keylaxavier01@gmail.com (K.V.M.X.); adrianneasilva@gmail.com (A.M.d.A.S.); acarol.luz@gmail.com (A.C.d.O.L.); 2 Federal Rural University of Pernambuco, UFRPE, Recife 52171-900, PE, Brazil; felipecabocloo@gmail.com; 3 Bioinformatics Core, Aggeu Magalhães Institute, IAM/Fiocruz, Recife 50740-465, PE, Brazil; biatoscano@gmail.com (B.S.T.d.M.); jlpitta82@gmail.com (J.L.d.L.P.P.)

**Keywords:** bacteriophages, antimicrobial resistance, CRISPR/Cas system

## Abstract

*Pseudomonas aeruginosa* is a major pathogen associated with hospital-acquired infections, and the spread of carbapenem-resistant isolates highlights the urgency of developing non-conventional therapies, such as phage therapy. For this alternative to be effective, understanding phage–host interactions is crucial for the selection of candidate phages and offers new insights into these dynamics. **Background/Objectives**: This study aimed to characterize prophage diversity in clinical *P. aeruginosa* genomes, assess the relationship between phages and the CRISPR/Cas system, and investigate the potential role of prophages in disseminating resistance genes. **Methods**: A total of 141 genomes from Brazilian hospitals were analyzed. Prophage detection was performed using VIBRANT, and in silico analyses were conducted to evaluate taxonomic diversity, the presence of resistance genes, phage life cycle, genomic distribution, and the presence of the CRISPR/Cas system. **Results**: A total of 841 viral sequences were identified by the VIBRANT tool, of which 498 were confirmed by CheckV, with a predominance of the class Caudoviricetes and high overall phage diversity. No statistically significant difference was observed in the number of prophages between isolates with and without CRISPR/Cas systems. Prophages carrying resistance genes such as *rsmA*, *OXA-56*, *SPM-1*, and others were detected in isolates harboring the type I-C CRISPR/Cas system. Additionally, prophages showed no preference for specific insertion sites along the bacterial genome. **Conclusions**: These findings provide evidence of a well-established phage–host relationship. The dual role of prophages—as vectors of antimicrobial resistance and as potential therapeutic agents—reflects their dynamic impact on bacterial communities and reinforces their importance in developing new strategies to combat antimicrobial resistance.

## 1. Introduction

Carbapenem resistance in Gram-negative bacteria is a global public health concern due to the increasing spread of resistance to this class of antimicrobials. Carbapenems are known for their broad-spectrum activity and are used as a last-resort treatment for severe infections [1]. In 2024, the WHO updated its list of priority pathogens associated with antimicrobial resistance, highlighting carbapenem-resistant Gram-negative bacteria as a top priority for the development of new therapies, as well as for control and prevention strategies [2].

*P. aeruginosa* has been categorized as a high-priority pathogen on the WHO list (2024) and is considered one of the main causes of healthcare-associated infections (HAIs), particularly affecting immunocompromised patients. Due to its intrinsic and acquired resistance to antimicrobials, as well as its ability to produce biofilms, therapeutic options for treating *P. aeruginosa* infections are limited [2,3].

One of the promising therapeutic approaches to address antimicrobial resistance is phage therapy, which involves the administration of bacteriophages—viruses that specifically target bacteria and are capable of killing bacterial cells. However, not all phages are suitable candidates for therapeutic use. Ideally, strictly lytic phages are preferred, as they lyse bacterial cells and directly combat the source of infection [4].

In contrast to strictly lytic phages, temperate (lysogenic) phages can follow an alternative pathway to bacterial lysis by integrating their genome into the bacterial chromosome, thereby behaving as a prophage and replicating along with the host cell. Under stressful conditions, the lysogenic phage may be induced to enter the lytic cycle. During this transition, the phage DNA excises itself from the bacterial genome, initiates replication, and ultimately leads to the production of new phage particles, resulting in the lysis of the host cell and the release of newly formed virions [5,6].

Bacteriophages and bacteria exhibit co-evolutionary dynamics similar to an arms race, in which both species must develop strategies for attack and survival. Within this process of modulation and adaptation, one proposed perspective is that phages, despite being intracellular parasites, may act as symbionts in their hosts due to the presence of phage-carried genes that enhance bacterial fitness [7].

In addition to genetic contributions, prophages can regulate the infection dynamics of related phages through a mechanism known as superinfection exclusion (SIE). This strategy allows prophages integrated into the bacterial genome to block subsequent infections by similar or identical phages, thereby ensuring their own maintenance within the host. SIE not only benefits the prophage but also has biological implications for the host bacterium, as it can provide temporary protection against infections by lytic phages, which might otherwise kill the host cell. Given this, the identification and characterization of prophages within bacterial genomes are essential considerations in the development of effective and safe phage therapies [8].

Moreover, to counter phage infection, bacteria have evolved adaptive immune systems, such as the CRISPR/Cas system (Clustered Regularly Interspaced Short Palindromic Repeats and CRISPR-associated genes), one of the most important antiphage defense strategies in bacteria. This mechanism provides acquired immunity by capturing fragments of invading phage DNA, which are then used to recognize and neutralize future infections by the same or similar phages. Upon detecting complementary sequences, the system recruits associated proteins, including nucleases, to cleave the foreign genetic material, thereby preventing successful infection [9,10].

For the proper implementation of phage therapy, in addition to understanding the mechanisms underlying phage–bacteria interactions, it is essential to identify the phages that interact with clinical *P. aeruginosa* isolates, their genomic features, and their role in modulating bacterial responses in the environment.

The approach described here analyzed clinical genomes of *P. aeruginosa* in search of putative phages and provided insights into the diversity of phages affecting this pathogen. We also characterized the taxonomy of the extracted viral sequences, explored the relationship between the presence of phages and the occurrence of the CRISPR/Cas system in *P. aeruginosa* genomes, investigated the chromosomal distribution of these phages within the host, their life cycle, and the detection of resistance genes carried by phages.

## 2. Materials and Methods

### 2.1. P. aeruginosa Genome

A total of 141 complete genomes of *P. aeruginosa* from clinical samples collected in hospitals across various Brazilian states (Pernambuco, Bahia, Ceará, Minas Gerais, Rio de Janeiro, São Paulo, Santa Catarina, Goiás, and Paraná) were selected from the NCBI database for this study. Of the 141 genomes analyzed in this study, 25 (from Recife-Pernambuco) were previously sequenced [11,12] and deposited in the NCBI under BioProject ID PRJNA514718. Additional epidemiological information on the genomes used, such as collection date, isolation source, location, MLST for species confirmation and the corresponding NCBI accession numbers, are available in Supplementary Material S1.

### 2.2. CRISPR System Identification

The identification of the CRISPR/Cas system in the genomes deposited in the NCBI database was performed using the standalone implementation of the CRISPRCasFinder tool [9]. The strains were then classified according to the type and subtype of CRISPR/Cas system present and categorized as CRISPR/Cas-positive if any type of system was detected, or CRISPR/Cas-negative otherwise.

### 2.3. Prophage Identification

The tool used for prophage identification was VIBRANT [13]. The results generated by VIBRANT were analyzed to identify phage sequences, their location within the host genome, and their length. Once the sequences were obtained, the identified phage regions were compared against previously deposited genomes in the NCBI Virus database to confirm the presence and identity of the phages. Alignments of unidentifiable regions were performed using the Mugsy tool [14].

The sequences extracted by VIBRANT had their quality assessed using CheckV (https://phage.usegalaxy.eu/, accessed on 27 April 2025), a specific pipeline for evaluating the quality of viral genomes. The sequences were classified as: Complete; High quality (>90% completeness); Medium quality (50–90% completeness); Low quality (<50% completeness); and Not determined.

### 2.4. Prophage Distribution in P. aeruginosa Genomes

The distribution of prophages across the bacterial genomes was analyzed based on the data extracted by VIBRANT. All positions were organized, recording the start and end points of each prophage, as well as their length. Subsequently, the data were mapped in a density plot generated in RStudio v4.4.0 using the ggplot2 package, specifically, with the functions geom_density and aes.

### 2.5. Clustering, Phylogeny, and Dendogram

The 841 phages identified in this study were mapped and grouped based on sequence similarity. Mapping was performed using the PhaMMseqs tool v1.0.4 [15]. However, due to the large volume of sequences and the substantial differences among them, the software was unable to operate under the standard clustering mode. To overcome this limitation, the parameter “—cluster-mode 1” was applied, enabling the single-linkage mode. This mode is more sensitive for detecting distant homologies, although it tends to generate a larger number of clusters.

The output file generated by PhaMMseqs v1.0.4 was then used as input for the PhamClust software v1.3.3 [16]. PhamClust was executed with default parameters, employing the Proteomic Equivalence Quotient (PEQ) metric to calculate the distance matrix.

Phylogenetic analysis was conducted using the VICTOR online server (https://victor.dsmz.de, accessed on 27 April 2025) [17]. All pairwise nucleotide sequence comparisons were performed using the Genome-BLAST Distance Phylogeny (GBDP) method [18] under the settings recommended for prokaryotic viruses.

The resultant intergenomic distances were utilized to generate a balanced minimal evolution tree with branch support using FASTME with SPR post-processing [19]. A total of 100 pseudo-bootstrap replicates were used to determine branch support. Trees were midpoint-rooted [20] and visualized using ggtree [21].

The OPTSIL program [22] was used to determine taxon boundaries at the species, genus, and family levels, following the recommended clustering thresholds and applying a F value (the fraction of links required for cluster fusion) of 0.5 [17].

The dendrogram of *P. aeruginosa* isolates was constructed based on categorical and quantitative data, including the type of CRISPR/Cas system, MLST profile, and the number of prophages identified per genome. The data matrix was organized in the RStudio V4.4.0 environment, and hierarchical clustering analysis was performed using the hclust(4) function, applied to a dissimilarity matrix generated with the dist(0, 1) function.qu

### 2.6. Identification of Resistance Genes and Virulence Factors in Prophages

The search for antibiotic resistance genes was performed using the Resistance Gene Identifier (RGI) (https://card.mcmaster.ca/analyze/rgi, accessed on 27 April 2025), a tool based on the Comprehensive Antibiotic Resistance Database (CARD) [23]. For the identification of virulence factors, the sequences were submitted to ABRIcate, available on the Galaxy EU (https://usegalaxy.eu/, accessed on 27 April 2025). Viral sequences positive for the presence of resistance and/or virulence genes were annotated using the Pharokka tool (https://phage.usegalaxy.eu/, accessed on 27 April 2025).

### 2.7. Prophage Life Cycle

To infer whether the bacteriophage life cycle was lytic or lysogenic, the PHACTS tool (PHAge Classification Tool Set) [24] was used. This computational tool is widely applied for classifying and predicting bacteriophage life cycles based on genomic and structural features. The analysis was performed by submitting the phage genomic data to PHACTS, which uses specific algorithms to identify genes and patterns associated with life cycle types. The results provided by PHACTS were interpreted based on the probabilities generated by its predictive model and were used as criteria for the final classification of the phage life cycle.

### 2.8. Statistical Correlations

Data regarding the number of phage regions per genome, categorized according to the presence or absence of the CRISPR/Cas system, were subjected to exploratory statistical analysis using the Jamovi software (https://www.jamovi.org). To evaluate the possible correlation between the number of viral regions and the presence of the CRISPR/Cas system, Spearman’s correlation test was applied. Only results with a *p*-value < 0.05 were considered statistically significant.

## 3. Results

### 3.1. In Silico Analysis Identifies Prophages in Brazilian Clinical Isolates of P. aeruginosa

A total of 141 *P. aeruginosa* genome sequences were selected and analyzed to investigate the frequency and distribution of prophages in Brazilian clinical isolates. Using the VIBRANT software to search for prophages within the 141 genomes, a total of 841 viral regions were identified, with 498 confirmed by CheckV. All genomes contained at least one sequence showing similarity to a phage (Supplementary Material S2).

A wide variation in the length of the viral sequences was observed. The largest sequence was 178,563 bp, and the smallest was 3028 bp. The average length of the regions was 31,873 bp, with a median of 29,641 bp.

To determine the prevalence of prophages in the *P. aeruginosa* genomes, the number of prophages per genome was analyzed. From this analysis, it was observed that some phages were rarely found, while others were more commonly present in the isolates’ genomes.

One prophage identified only once showed high sequence similarity to *Pseudomonas phage* PAJU2. In contrast, *Pseudomonas phage phi3* was detected 50 times in 32 analyzed *P. aeruginosa* genomes. It is worth noting that the phages classified as “*Bacteriophage* sp.” and “*Caudoviricetes* sp.” were the most frequently observed. However, it was not possible to determine significant similarity with previously characterized phages (Table 1).

The completeness and quality of the viral sequences were assessed using CheckV. Among the genomes analyzed, 60% were classified as high or medium quality, while 39% were of low quality (completeness < 50%) (Supplementary Material S3).

Only 1% of the sequences (11 in total) were classified as “not determined”. Despite the low completeness observed in some sequences, all were retained for subsequent analyses, as VIBRANT detected virus-associated proteins, indicating their viral potential. Moreover, host genome contamination exceeded 50% in only 3% of the sequences, suggesting minimal host interference in most cases. After this screening, the sequences were submitted to PhamCluster for group clustering. The clustering results are discussed later.

When classifying the samples as CRISPR/Cas-positive or negative, it was observed that the positive samples contained more viral regions than the negative ones, with an average of six regions compared to an average of five regions in the negative group. However, this difference was not significant, as evidenced by a p-value greater than 0.05 (Table 2).

The number of prophages identified varied markedly among the analyzed genomes. Some strains, such as Pae113 (CRISPR/Cas-positive) and UFMG-H10 (CRISPR/Cas-negative), harbored only a single prophage. In contrast, others contained a much higher number of distinct prophages, with the CRISPR/Cas-positive strain CCBH28612 carrying 17 unique viral sequences and the CRISPR/Cas-negative strain H2-9me containing 16 (Table 3). These results demonstrate both the coexistence of multiple distinct prophages within individual *P. aeruginosa* genomes and the high variability in prophage content across different strains.

**Figure 1:**
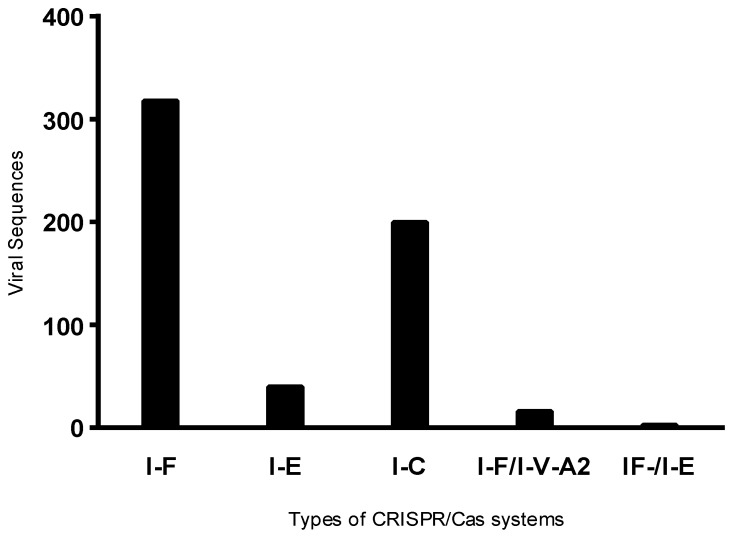
Distribution of viral regions according to CRISPR/Cas system types in positive bacterial isolates. Viral regions were grouped based on the CRISPR/Cas system type present in each isolate.

Of the 841 viral sequences observed, 587 were identified in isolates positive for the CRISPR system. Upon analyzing the frequency of these sequences by system type, phages were more frequently detected in hosts harboring the Type I-F system (Figure 1), which may be associated with the higher number of isolates positive for the I-F type. Furthermore, an average of four prophages per isolate was observed in Type I-F positive isolates, three in isolates harboring the I-E system, and seven in those with the I-C system.

**Figure 2:**
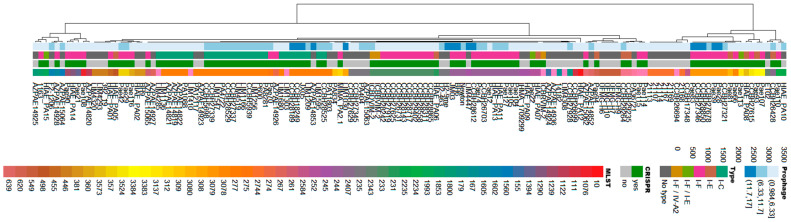
Hierarchical clustering dendrogram illustrating the genomic relationships among *P. aeruginosa* isolates. The clustering was performed based on three main features: the type of CRISPR/Cas system present, the multilocus sequence typing (MLST) profile, and the number of prophages identified in each genome. This analysis highlights patterns of similarity and divergence among the isolates according to these genetic features.

A dendrogram was constructed to demonstrate the relationship among *P. aeruginosa* isolates regarding the type of CRISPR/Cas system, MLST profile, and the number of prophages per genome. Figure 2 shows the distribution of the isolates in relation to these genetic characteristics. It was observed that the clusters were formed in a heterogeneous manner with respect to the isolates, but showed internal homogeneity regarding the analyzed characteristics (Figure 2).

### 3.2. Caudoviricetes Is the Most Commonly Identified Class of Prophages in P. aeruginosa Genomes

The recovered phage sequences were subjected to taxonomic classification. Of the 841 regions analyzed, 12 (1.4%) could not be taxonomically assigned due to a lack of significant similarity or because they exhibited low query coverage (<30%) against viral genomes previously deposited in the NCBI viral sequence database (www.ncbi.nlm.nih.gov, accessed on 27 May 2025). Among these 12 sequences, 6 were identified as three distinct pairs. For each pair of aligned sequences, both corresponded to the same phage and were identified in two different isolates. The remaining six unpaired sequences did not align with any others and were therefore considered as unique phages specific to their respective host genomes.

Among the 829 remaining viral regions, 140 were classified as bacteriophages with no similarity to any previously characterized phage; 136 were identified as *Caudoviricetes* sp., 14 as *Inoviridae* sp., and 1 as *Microviridae*. The remaining 538 sequences were grouped into 103 distinct phages, resulting in a total of 107 phage types based on their taxonomic classification.

**Figure 3:**
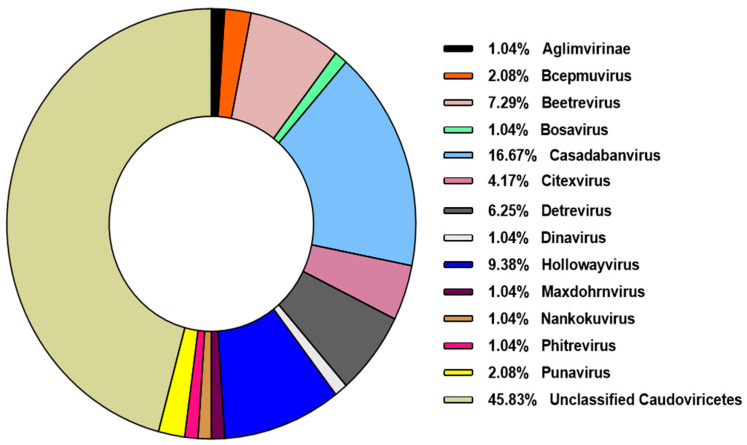
Prevalence of genera belonging to the class *Caudoviricetes*. The identified genera belonging to this class were classified according to their frequency among the analyzed viral sequences.

The majority of phage types (104 out of 107, 98%) were identified as belonging to the class Caudoviricetes. This class encompasses all tailed bacteriophages and represents the largest group of phage sequences deposited in the NCBI database. In 2022, with the updated classification system proposed by the International Committee on Taxonomy of Viruses (ICTV), morphology-based taxonomy was abolished, and the former order Caudovirales—along with the families Myoviridae, Podoviridae, and Siphoviridae—was reclassified under the broader class Caudoviricetes [25]. Among the Caudoviricetes phages identified, 44 (42.3%) could not be resolved to the genus level. However, the remaining sequences were distributed across 13 distinct viral genera, with Casadabanvirus being the most frequently represented (Figure 3).

The second most frequently identified class of prophages was Faserviricetes, accounting for approximately 2.6% (*n* = 4) of the total. Members of the Faserviricetes class are flexible filamentous phages with a positive-sense single-stranded DNA genome, such as those belonging to the family Inoviridae, exemplified by the phage Primolicivirus Pf1 [26]. The third most common class, albeit representing only 0.7% (*n* = 1) of all identified prophage genomes, was Malgrandaviricetes, which includes phages from the family Microviridae. These viruses possess an icosahedral morphology and ssDNA genomes and primarily infect enterobacteria [26]. Viral sequences that could not be taxonomically classified represented 1.9% of the dataset.

To gain a better understanding of the sequences generically classified as “Bacteriophage” and “Caudoviricetes”, a comparative genomics analysis was performed using the PhamClust software v1.3.3. This analysis resulted in the formation of 91 clusters (Supplementary Material S4). Among the 140 sequences labeled as “Bacteriophage”, 114 were grouped into 15 distinct clusters, suggesting the presence of 15 potential phage types infecting *P. aeruginosa*. The remaining 26 sequences showed no detectable similarity to each other. Of the 136 sequences identified as “Caudoviricetes”, only 48 were distributed into 16 clusters, while the remaining regions were not clustered.

For each cluster formed, a representative sequence was selected to better understand the phylogenetic distribution of the phages classified as Bacteriophage and Caudoviricetes. This selection was based on the sequence with the highest similarity among the sequences within each cluster.

**Figure 4:**
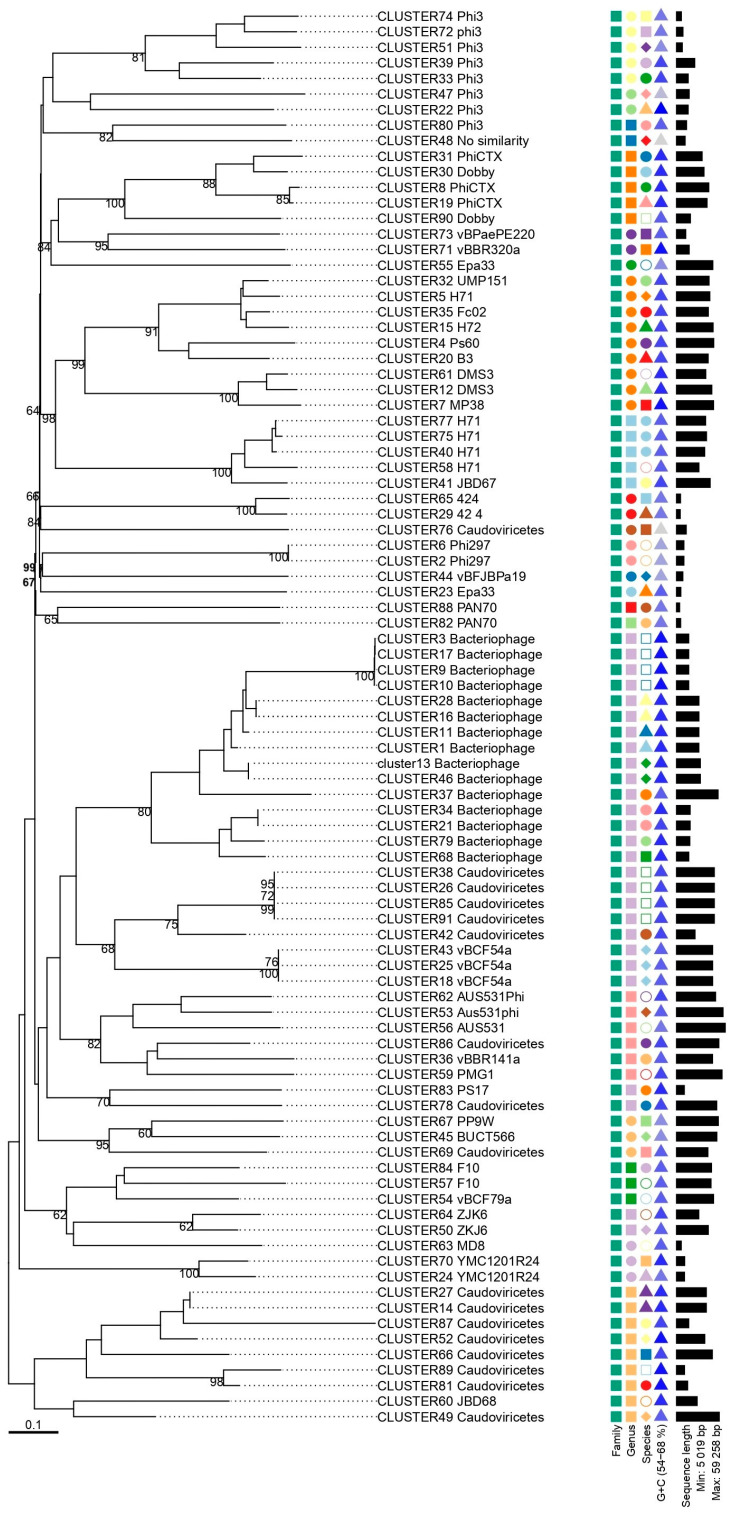
Phylogenetic analysis of representative prophages identified in clinical isolates of *P. aeruginosa*. The phylogenetic tree was constructed using the Genome-BLAST Distance Phylogeny (GBDP) method through the VICTOR platform, with 100 pseudo-bootstrap replicates to estimate branch support. For the additional phylogenetic features, families are represented by squares, indicating that all sequences belong to the same family. At the genus level, different geometric shapes are used to reflect greater diversity among the sequences: identical shapes and colors indicate the same genus, while different shapes and colors indicate different genera. GC content is represented by triangles, following the same logic—identical colors indicate the same GC content.

Based on the phylogenetic analysis, four major branches can be observed, with a distribution of smaller subgroups, highlighting the diversity of the *P. aeruginosa* phage community (Figure 4).

The sequences classified as Bacteriophage and Caudoviricetes are homogeneously distributed within their respective groups. Clusters corresponding to phages with already-defined taxonomy were grouped more individually and appeared distant from the unclassified sequences. Despite the formation of clusters, it was not possible to infer deeper evolutionary relationships for the unknown sequences.

### 3.3. P. aeruginosa Prophages Do Not Exhibit Unique Insertion Sites

To determine whether prophages, regardless of their taxonomic classification, are integrated into specific regions of the bacterial genome or are randomly distributed throughout the host DNA, all prophage insertion sites were mapped and plotted against a reference genome. This approach allowed for a comprehensive visualization of the genomic distribution of all analyzed prophages

**Figure 5:**
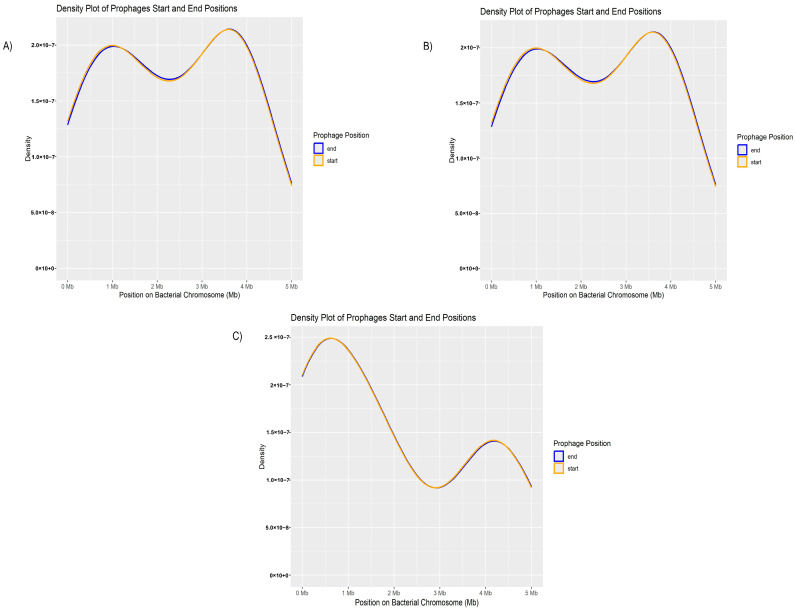
Distribution of prophages identified in clinical isolates of *P. aeruginosa*. (**A**) Map showing the complete distribution of all prophages extracted from the genomes of clinical isolates of *P. aeruginosa*, highlighting their distribution along the genome. (**B**) Detailed representation of the location and distribution of phage Phi3 along the chromosome of *P. aeruginosa*, with the orange line indicating the start of the phage sequence and the blue line indicating the end. (**C**) Detailed representation of the location and distribution of phage Phi297 along the chromosome of *P. aeruginosa*, with the orange line marking the start of the phage sequence and the blue line marking the end.

No concentration in specific genomic regions was observed. Based on the density plot (Figure 5A), the start and end positions of the prophages along the bacterial chromosome showed a continuous and dispersed distribution across the genome, suggesting that rather than integrating into preferential loci, prophages may be inserted at various sites throughout the host DNA, indicating a random pattern of genomic dispersion.

Although this result represents the distribution of all prophages, the genomic positions of the most frequently identified prophages were individually analyzed within the bacterial genome. First, the distribution of phage phi3 was assessed (Figure 5B).

The distribution of phage phi3 also shows overlapping density curves, indicating a continuous distribution along the bacterial chromosome. However, notable peaks are observed around 1 Mb and 4 Mb, suggesting a slight preference for insertion in these regions. Overall, the insertions appear to be broadly dispersed throughout the chromosome. The second phage analyzed was *phi297*. In this case, the distribution of the phage shows peaks at approximately 1 Mb and 3.5 Mb but similarly exhibits a relatively uniform distribution across the bacterial genome (Figure 5C).

Thus, based on the relative insertions of the phages within the genome, a continuous dispersion was observed, with a less directed or more random pattern.

### 3.4. Presence of Antibiotic Resistance Genes in Viral Sequences

A search for antibiotic resistance genes was conducted in viral genomes, given that these viruses have the ability to mediate the transfer of resistance genes between different bacterial species, thus contributing to the rapid spread of antibiotic resistance. Among the 841 viral sequences analyzed, derived from 141 bacterial genomes, resistance genes were identified in 19 viral sequences (1.6%), which were distributed across 8 bacterial genomes (5.7%) (Table 4).

When evaluating viral sequences positive for the presence of resistance genes, we considered only those containing ARGs located within the phage region. Genes located in the terminal regions of the sequences, although detected by CARD, were not considered positive resistance genes in the analysis, as they may be associated with the host genome. Importantly, none of the retained ARGs were located within the direct terminal repeat (DTR) regions.

To confirm the viral origin, sequences that were positive for these genes were annotated and showed typical phage features, confirmed by the presence of hallmark genes such as capsid, holin, and phage tail tube. Although CheckV classified these sequences as “low quality”, we retained them in the analysis due to the consistent presence of multiple phage structural genes and confirmation through Pharokka annotation (Supplementary Material S5). The presence of genes encoding proteins from various classes associated with antimicrobial resistance was observed. Notably, these include β-lactamases (*blaOXA-56*), genes linked to efflux pump systems (*cmx*), and sulfonamide resistance genes (*sul1*) [27]. No virulence genes were detected.

**Figure 6:**
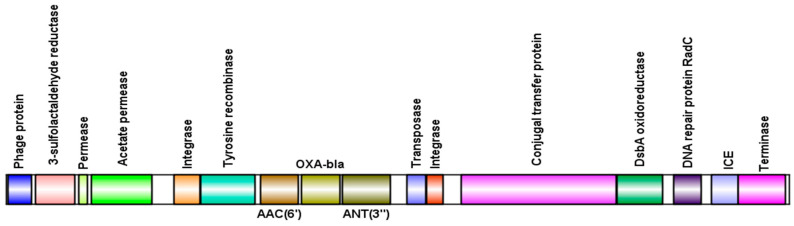
Schematic representation of resistance genes in gene cassettes (*aacA4*, *aadA7*, and *blaOXA-56*) present in a viral sequence, including their adjacent genes.

Isolates that are positive for phages carrying the *blaOXA-56* gene belong to sequence type ST277 and harbor a type I-C CRISPR/Cas system. This clone is characterized by an expanded resistance profile, including the presence of class 1 integron In163, which organizes the *aacA4*, *aadA7*, and *blaOXA-56* genes into gene cassettes [28,29]. Notably, in isolate LIM1410, the inserted prophages exhibited this same gene association, harboring the *aacA4*, *aadA7*, and *blaOXA-56* genes simultaneously (Figure 6). 

### 3.5. The Lytic Cycle Is More Frequent in Clinical Isolates of P. aeruginosa

Using PHACTS, a total of 841 prophage sequences from different bacterial genomes were analyzed to infer their potential behavior—whether they are likely to enter the lytic cycle or remain in the lysogenic state. Among these sequences, 474 were classified as prophages with a higher potential to initiate the lytic cycle, while 367 showed a higher tendency to maintain the lysogenic cycle (Supplementary Material S6).

Regarding the prophages carrying resistance genes, 13 exhibited a lysogenic life cycle, while 23 were identified as lytic phages.

The presence of 474 prophages with lytic potential suggests a significant capacity of these genetic elements to excise themselves from the host genome and enter the lytic cycle. This potential enables rapid viral replication in host-rich environments, efficient dispersal through cell lysis, and a selective advantage in competitive conditions. In contrast, the 367 prophages with lysogenic potential indicate an adaptive strategy that favors long-term persistence, horizontal gene transfer, and evasion of host immune defenses.

The coexistence of prophages with both lytic and lysogenic potential reflects a complex interaction between phages and their host populations, influenced by environmental and genetic factors. Variables such as bacterial density, nutrient availability, and the presence of abiotic stressors can modulate the predominance of one cycle over the other, thereby shaping the ecology and evolution of microbial communities [30,31].

## 4. Discussion

The diversity of phages within bacterial ecosystems is vastly greater than the number of genomes currently deposited in public databases. Studies focused on the identification of prophages and metagenomic analyses can substantially increase the number of available sequences, while also providing deeper insights into this reservoir of phage genetic diversity and their respective hosts [32].

Although the recovery of prophage sequences from bacterial genomes does not allow for the isolation of viable phage particles—as is possible with conventional culture-based methods—the data obtained through in silico analyses enable valuable discoveries and foster meaningful discussions about this viral community.

Despite the vast viral diversity, our taxonomic data support and reinforce the evidence that the class *Caudoviricetes* is predominant among phages associated with different bacterial communities, underscoring its relevance in regulating bacterial populations and shaping microbial community structure [33,34,35].

In the present analysis, more than 280 prophage sequences showed no homology with previously described phages. Nonetheless, it was possible to group them and analyze the occurrence of uncharacterized phages in Brazilian *P. aeruginosa* isolates. Most prophages integrate into the host bacterial genome through site-specific recombination at att sites or by transposition into random locations. These insertions usually occur in genomic regions that cause minimal harm to the host, such as areas near non-coding genes like tRNA, regions with few functional genes, or intergenic regions [36].

Phage therapy offers advantages over antibiotics, especially in the phages’ ability to coevolve with bacteria. In fact, unlike antibiotics—which can quickly become ineffective due to bacterial resistance—phages can coevolve in situ with their hosts, potentially making them more effective in the long term. Although the phenomenon of overexpression indeed poses a challenge at this stage, the combination of effective antibiotics with the selection of suitable phages for phage therapy has shown promise in tackling infections caused by pathogens [8,37].

Phages play a dynamic role in bacterial evolution, going beyond the mere transfer of resistance genes. The interaction between phages and their bacterial hosts can be viewed as a true “biological warfare”, in which bacteria develop defense mechanisms such as CRISPR/Cas systems that provide adaptive immunity against viral infections. In addition, restriction-modification (RM) systems also contribute to bacterial resistance by enabling the recognition and degradation of invading phage DNA through restriction endonucleases [10,38].

Phages themselves can exert control over related phage infections through superinfection exclusion (SIE), a mechanism by which an integrated prophage prevents infection by similar phages. Interestingly, a study based on the genomic analysis of phages using the Anderson phage typing scheme revealed a diversity in phage–host interactions, including the absence of known anti-CRISPR genes, highlighting new opportunities for exploring phage–host–phage relationships [10].

Understanding these processes is essential for the development of novel therapeutic approaches involving phages or phage inhibitors, enhancing efforts to combat antimicrobial resistance. Furthermore, the prevalence and diversity of phages in hospital environments—where selective pressure is intense—may amplify the dissemination of resistance genes, thereby compromising the effectiveness of conventional treatments.

Our findings suggest that *P. aeruginosa* phages integrate in a non-targeted manner into the host genome, or at multiple integration sites throughout the genome. It is important to highlight that each type of insertion may confer a specific advantage to the bacterium. The dispersed presence of prophages enables rapid adaptation of the bacterial host, as they function as genetic buffer zones, promoting genetic variability within the bacterial population.

This variability can accelerate adaptation to environmental changes and selective pressures. On the other hand, such random insertions may also be more deleterious, which is why site-specific insertions tend to confer greater genetic stability to the host. Thus, each bacterium is likely to adopt different strategies for interacting with prophages, depending on its environment, ecological niche, and the selective pressures it encounters [36,39].

Bacteria develop resistance to antimicrobials through intrinsic processes—via the expression of genes located on their own chromosome—or through acquired mechanisms, including mutations or the acquisition of resistance genes via Horizontal Gene Transfer (HGT). Phages are capable of transferring genetic material from one bacterium to another through transduction, including antimicrobial resistance genes, highlighting the role of phages in the acquisition and dissemination of ARGs (Antimicrobial Resistance Genes) [40].

Our results revealed the presence of phages carrying resistance genes. Previous studies involving other clinically relevant bacterial species, such as *Staphylococcus aureus* and *Escherichia coli*, have demonstrated that these viral elements can encapsulate fragments of bacterial DNA and, through transduction, transfer this material from donor to recipient cells [41,42].

Although this process may be less efficient—particularly in the presence of virulent phages that lyse co-infected cells—and given that mechanisms such as conjugation are more frequently employed by bacteria, the role of phages in modulating the bacterial genome remains significant and should not be underestimated [41,43].

When evaluating the sequences of prophages, CheckV returned a low completeness quality of the sequences; however, this value does not disregard that prophages can act as vectors. Although the identification of antibiotic resistance genes (ARGs) in phage genomes should be performed with caution, here we demonstrate that even in low-quality sequences, it was possible to detect ARGs associated with viral elements.

Most of the viral sequences positive for resistance genes are associated with the genomes of isolates belonging to ST277. This clone is considered endemic among Brazilian strains of *P. aeruginosa* and is characterized by a high-level antibiotic resistance profile and by its association with the type I-C CRISPR/Cas system, which is frequently located within genomic island regions [12,28,44].

Genomic islands are DNA segments acquired through horizontal gene transfer, usually derived from mobile genetic elements or phages. In *P. aeruginosa*, these segments are known as PAGIs (*Pseudomonas aeruginosa* Genomic Islands) and commonly harbor genes that provide adaptive advantages to the host, such as antibiotic resistance, virulence factors, and genes associated with metabolism [29,44].

To understand how phages carry resistance genes associated with class 1 integron In163 (*aacA4*, *aadA7*, and *blaOXA-56*), the genome of phage positive for these genes were annotated. The analysis revealed the presence of typical phage proteins, confirming their viral identity. Additionally, in the phage associated with the *aacA4*, *aadA7*, and *blaOXA-56* genes, proteins related to integrative conjugative elements were identified. These findings suggest that, during the viral assembly process, these phages may have incorporated bacterial sequences into their genomes, potentially including genes or elements similar to Integrative and Conjugative Elements (ICEs).

Furthermore, do Nascimento et al. (2020) [45] characterized PAGIs enriched with transposases, integrases, and phage-associated proteins, along with the prevalence of resistance genes such as *blaSPM-1*, *blaOXA-56*, *rmtD*, *cmx*, and *sul1*. Based on the data obtained and the annotation of the genomic sequences, a significant association was observed between the presence of phages and the ability of *P. aeruginosa* ST277 to acquire and disseminate antimicrobial resistance [45].

The survey of prophages inserted in clinical *P. aeruginosa* genomes in Brazil revealed a prevalence of phages belonging to the class *Caudoviricetes*, reflecting the high diversity of phages within bacterial communities. The distribution of prophages throughout the host genome, without a preferential integration site, suggests a complex dynamic of viral integration with important implications for bacterial evolution. Furthermore, the presence of antibiotic resistance genes encoded by prophages highlights their role as vectors in the dissemination of resistance determinants, posing a new challenge for the control of bacterial infections.

## 5. Conclusions

The survey of prophages inserted in clinical *P. aeruginosa* genomes in Brazil revealed a prevalence of phages belonging to the class Caudoviricetes, reflecting the high diversity of phages within bacterial communities. The distribution of prophages throughout the host genome, without a preferential integration site, suggests a complex dynamic of viral integration with important implications for bacterial evolution. Furthermore, the presence of antibiotic resistance genes encoded by prophages highlights their role as vectors in the dissemination of resistance determinants, posing a new challenge for the control of bacterial infections. Understanding this viral diversity highlights that prophages not only coexist with their hosts but also play important roles in bacterial evolution and the dissemination of resistance genes. Grasping this relationship is essential to interpret the events that shape bacterial genomic plasticity and, consequently, their adaptation to selective environments. Moreover, this knowledge contributes to the development of more effective strategies for monitoring and controlling antimicrobial resistance, as well as advancing phage-based therapeutic approaches as promising alternatives to traditional antibiotics.

## Figures and Tables

**Table 1:** Most and least frequent prophages identified in *P. aeruginosa* strains.

Most Common Prophages		Less Common Prophages	
Phage	Total	Phage	Total
Bacteriophage sp.	147	Pseudomonas phage PAJU2	1
Caudoviricetes sp.	136	Escherichia phage P1	1
Pseudomonas phage phi3	50	Pseudomonas phage JBD26	1
Pseudomonas phage phi297	35	Pseudomonas phage PA8	1
Pseudomonas phage H71	32	Pseudomonas phage JBD5	1
Pseudomonas phage Dobby	25	Burkholderia phage phiE255	1
Pseudomonas phage AUS531phi	24	Pseudomonas phage vB_Pae_CF125a	1
Pseudomonas phage phiCTX	22	Ralstonia phage Dina	1
Pseudomonas phage vB_Pae_CF54a	16	Pseudomonas phage D3112	1
Pseudomonas phage UMP151	15	Pseudomonas phage MP42	1

**Table 2:** Statistical data on the distribution of phage-designated regions in the studied *P. aeruginosa* genomes.

Group Description						
	Group	N	Mean	Median	Standard Deviation	Standard Error
Phage Region Count	Positive	93	6.31	6	3.64	0.377
	Negative	48	5.29	5	3.64	0.525
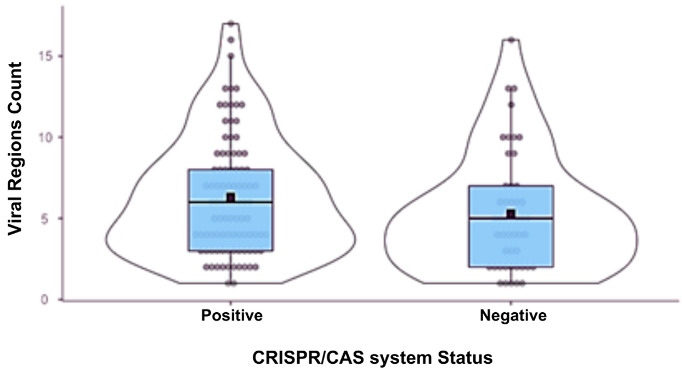						
Correlation matrix						
					**Viral region count**	
CRISPR/Cas status		Spearman’s rho			0.150	
		*p*-value			0.076	

**Table 3:** *P. aeruginosa* isolates with the highest and lowest number of identified prophages.

Highest Number of Prophages Identified		Fewest Number of Prophages Identified	
Isolate	Nº Prophages	Isolate	Nº Prophages
CCBH28612	17	AZPAE15065	1
H2-9me	16	ET02	1
Pae28	16	Pae113	1
Pae39	15	UFMG-H6	1
AZPAE14853	13	UFMG-H7	1
CCBH27346	13	UFMG-H9	1
JM03	13	UFMG-H10	1
LIM1030	13	Pae93	2
Pae83	13	Pae94	2
BH6	12	Pae110	2

**Table 4:** Antibiotic resistance genes observed in prophages of *P. aeruginosa* genomes.

Isolate	Start Position	Stop Position	Phage Lenght	CheckV	Resistence Gene
CCBH28529	5516	6358	28,935	Low-quality	*sul1*
CCBH28529	55,961	56,704	69,697	Low-quality	*rmtD*
CCBH28850	7614	8111	58,067	Low-quality	*dfrA21*
CCBH28850	8639	9478	58,067	Low-quality	*sul1*
JX05	4041	5216	62,157	Low-quality	*sul1*
JX05	6351	7190	62,157	Low-quality	*sul1*
JX05	9396	10,277	62,157	Low-quality	*cmx*
JX05	12,157	12,954	62,157	Low-quality	*aadA7*
JX05	13,016	13,816	62,157	Low-quality	*oxa-56*
LIM1030	2050	2889	41,494	Low-quality	*sul1*
LIM1030	2050	2889	41,494	Low-quality	*sul1*
LIM1166	7547	8722	29,997	Low-quality	*cmx*
LIM1166	7547	8722	29,997	Low-quality	*cmx*
LIM1410	49,583	50,215	178,563	Low-quality	*oxa-56*
LIM1410	50,296	51,096	178,563	Low-quality	*aac(6′)-Ib9*
LIM1410	51,158	51,955	178,563	Low-quality	*aadA7*
LIM1547	1263	2174	9701	Low-quality	*sul1*
LIM1547	5140	6315	9701	Low-quality	*cmx*
LIM4519	7397	8572	12,669	Low-quality	*cmx*

## Data Availability

The original contributions presented in the study are included in the article, further inquiries can be directed to the corresponding author.

## Supplementary Materials

The following supporting information can be downloaded at: https://www.mdpi.com/article/10.3390/genes16060656/s1, Supplementary Material S1: Epidemiological information on the 141 Pseudomonas aeruginosa isolates; Supplementary Material S2: Data on the 841 prophages extracted from VIBRANT, including information on the closest phage, length, and location; Supplementary Material S3: Results obtained through CheckV. Supplementary Material S4: Clusters generated from the analysis performed with PhamClust, grouping sequences based on functional and structural similarity; Supplementary Material S5: Annotation and data related to resistance genes observed in viral sequences; Supplementary Material S6: Life cycle identified by PHACTS for the 841 viral sequences studied.

## Abbreviations

CRISPR/CAS	Clustered Regularly Interspaced Short Palindromic Repeats and CRISPR-associated genes
DNA	Deoxyribonucleic acid
ICEs	Integrative and Conjugative Elements
PAGIs	*Pseudomonas aeruginosa* Genomic Islands
